# Editorial: Current concepts related to the understanding of femoroacetabular impingement syndrome and advancements in perioperative arthroscopic management: An update

**DOI:** 10.3389/fsurg.2022.1004975

**Published:** 2022-09-14

**Authors:** Shane J. Nho, Morgan Rice

**Affiliations:** Rush University Medical Center, Department of Orthopaedic Surgery, Division of Sports Medicine, Section of Young Adult Hip Surgery, Chicago, IL, United States

**Keywords:** femoroacetabular impingement, hip arthroscopy, motion analysis, hip labral repair, cam deformity, pincer deformity, hip morphology

**Editorial on the Research Topic** Current concepts related to the understanding of femoroacetabular impingement syndrome and advancements in perioperative arthroscopic management: An update By Nho SJ and Rice M. (2022) Front. Surg. 9: 1004975. doi: 10.3389/fsurg.2022.1004975

Femoroacetabular Impingement Syndrome (FAIS) is a pathologic entity defined by abnormal contact forces in the hip joint secondary to morphologic changes involving the femoral head–neck junction or acetabulum rim. Abnormal forces lead to the development of acetabular chondral lesions, labral tears and progressive joint damage. Thus, early recognition and treatment of FAIS is critical to prevent secondary pathologies, such as osteoarthritis of the hip. The 3-Dimensional kinematic study by Malloy et al. will show how hip arthroscopic surgery for FAIS affects the planar covariation strategy of the hip joint during walking can provide insight as to whether joint function improves after surgery, and to determine if continued functional deficits may persist.

FAI has been categorized into three different subtypes: cam impingement, pincer impingement, and combined cam and pincer impingement with the most common etiology by far being cam impingement. Although relatively rare in the general population, the retrospective diagnostic study by Hanzlik et al. will show the prevalence of osseous cam deformity in a cross section of a general population may be significantly higher than previously reported. In treating FAIS, the systematic review by Bessa et al. will show how arthroscopic autograft reconstruction of the acetabular labrum results in significant improvement in the short- and mid-term patient reported outcomes.

The remaining articles look beyond early recognition in attempts to identify factors that contribute to the development of FAIS even before structural deformity is clinically apparent. The systematic review by Clapp et al. examines the natural history of joint hyperlaxity on the development of FAIS. The review also discusses how resulting capsular laxity may result in more complications, revisions and poorer outcomes after treatment for subsequent FAIS. The case series by Knapik et al. Highlights how extra-articular disease may also contribute to poorer outcomes after treatment for FAIS. The study proposes a new anatomic classification system based on anatomic appearance as a means of providing a simpler method of classifying anterior inferior iliac spine (AIIS) dysmorphisms, the second most common cause of failed hip preservation surgery requiring revision behind insufficient FAI resection.

The editors of the Current Concepts Related to the Understanding of Femoroacetabular Impingement Syndrome and Advancements in Perioperative Arthroscopic Management: An Update research topic believe that the content provides the most up to date information in a field that is rapidly evolving. We hope that you find these articles enjoyable and stimulating of further discussion and understanding of FAIS.

